# Paroxysmal Sympathetic Hyperactivity in Non-traumatic Brain Injury: A Retrospective Analysis From the Intensive Care Unit of a Tertiary Care Academic University

**DOI:** 10.7759/cureus.109022

**Published:** 2026-05-17

**Authors:** Jitendra S Chahar, Sangam Yadav, Sai Saran, Mohan Gurjar, Afzal Azim, Banani Poddar

**Affiliations:** 1 Critical Care Medicine, Sanjay Gandhi Postgraduate Institute of Medical Sciences, Lucknow, IND

**Keywords:** clonidine, hypoxic-ischemic encephalopathy, meningoencephalitis, non-traumatic brain injury, paroxysmal sympathetic hyperactivity, post-cardiac arrest encephalopathy, propranolol, psh, psh-am score

## Abstract

Background: Paroxysmal sympathetic hyperactivity (PSH) can occur in comatose patients with a history of non-traumatic brain injury. In the intensive care unit, it is very challenging to diagnose such a condition, as there are a lot of masquerades.

Methods: This was a retrospective study, which screened case records of adult patients (age > 18 years), referred to a 26-bed intensive care unit of a tertiary care academic university, admitted with a comatose state, either post-cardiac arrest status with presumptive diagnosis of hypoxic ischemic brain injury, or patients with suspected meningo-encephalitis for a period of one year. Patient case files suggestive of a history of traumatic brain injury, stroke, epilepsy, and autonomic dysfunction were excluded. Case files with a Paroxysmal Sympathetic Hyperactivity-Assessment Measure (PSH-AM) score ≥17 were included. The case files were reviewed for a period of two weeks starting from the day PSH was diagnosed, and the outcome was assessed in terms of changes in PSH-AM scores and Glasgow Outcome Scale Extended (GOS-E) at ICU discharge.

Results: The prevalence of PSH was 17% in comatose patients with hypoxic ischemic encephalopathy (n = 04) and acute meningo-encephalitis (n = 03). There were seven patients with a diagnosis of probable PSH (PSH-AM score ≥17). The median age of the study cohort was 41 years (35-48 years). The median value of severity of illness at ICU admission as assessed by the Acute Physiology and Chronic Health Evaluation II (APACHE II) was 19 (13-21). The median PSH-AM score was 20 (18-21) at the time of diagnosis of PSH, which was 38 days (21-42 days) from the neurological insult. The PSH score reduced to 5 (3-14) after two weeks of treatment (p < 0.001) with propranolol and clonidine. The median GOS-E of the study population was 2 (2-3).

Conclusion: A high index of suspicion should be kept for diagnosing PSH in patients with non-traumatic brain injury, particularly those with hypoxic brain injury and encephalitis. Episodic tachycardia, hypertension, diaphoresis, and posturing are important features to suspect PSH in comatose patients with a history of non-traumatic brain injury after ruling out other mimics in the ICU, such as sepsis. The PSH-AM score can be helpful in screening for PSH in such patients. Early identification and management of PSH in such critically ill patients can reduce the number of medical interventions.

## Introduction

Paroxysmal sympathetic hyperactivity (PSH) was a known entity with different names from the 1930s, such as diencephalic autonomic epilepsy, autonomic seizures, subcortical epilepsy, etc. [1]. After decades of work, the consensus labelled the syndrome as “paroxysmal,” as the symptoms occur in bursts intermittently, “sympathetic,” as this is the system that overdominates [2,3]. The diagnosis of PSH is made based on the presence of recurrent episodes of sympathetic overactivity characterized by tachycardia, hypertension, hyperthermia, tachypnoea, diaphoresis, and abnormal posturing. Exclusion of alternative diagnoses such as sepsis, past history of epilepsy, or drug withdrawal is an important component [4]. There are many postulated hypotheses for this, such as the loss of inhibitory signals from the cortex and brain stem to the spinal cord due to shear stress, and thereby minor stimuli such as urine retention, mobilization, tactile stimuli, etc., trigger massive release of catecholamines like norepinephrine, leading to sympathetic storms. Excitatory-inhibitory ratio (EIR theory), which is an imbalance between excitatory and inhibitory processes of the nervous system and the neuro-endocrine system, provides insight into the heightened activation of the hypothalamic-pituitary axis (HPA) [5-9].

Historically, the PSH-Assessment Measure (PSH-AM) score was used to support the diagnosis of PSH [6]. Based on the PSH-AM score, the probability of PSH is categorized as follows: a score <8 indicates PSH is unlikely, 8-16 indicates possible PSH, and ≥17 indicates probable PSH. Diagnosing this condition in the ICU is challenging, as symptoms are often masked by sepsis, drug withdrawal, or deep vein thrombosis [5]. PSH is typically seen in individuals who have suffered traumatic brain injury (TBI), particularly following diffuse axonal injury, where it may affect as many as 15-30% of patients. Such a condition has also been described in comatose patients with a history of non-TBI [5-10].

This retrospective study was done in the ICU of a tertiary care academic university, with the primary objective of the study being to determine the prevalence of probable PSH (defined as PSH-AM score ≥17) in comatose patients with non‑TBI (hypoxic ischemic encephalopathy or encephalitis). The secondary objective of the study was to evaluate the clinical course, treatment response, and ICU outcomes of these patients using PSH‑AM scores and Glasgow Outcome Scale Extended (GOS-E).

## Materials and methods

Study design, setting, and patients

This retrospective study screened case records of adult patients (age >18 years) admitted with either post-cardiac arrest status with return of spontaneous circulation, without intact neurological function, with presumptive diagnosis of hypoxic ischemic brain injury, or patients with suspected meningo-encephalitis. These patients were referred to a 26-bed intensive care unit of a tertiary care academic university in northern India from October 2024 to September 2025. Patient case files suggestive of a history of TBI, stroke, epilepsy, and autonomic dysfunction were excluded. The PSH-AM score was used to support the diagnosis of PSH [6]. The PSH-AM score consists of two components: the clinical feature scale (CFS) (Table 1) and the diagnosis likelihood tool (DLT) (Table 2).

**Table 1:** Clinical feature scale (CFS) score Maximum CFS score [6] = 18; Severity of CFS score: 0 = nil; 1-6 = mild; 7-12 = moderate; ≥13 = severe. Credit: Information in this table was derived from the original publication of the Paroxysmal Sympathetic Hyperactivity Assessment Measure (PSH-AM) score [6], published under the Creative Commons License.

Clinical feature	0	1	2	3
Heart rate (beats/min)	<100	100-119	120-139	≥140
Respiratory rate (breaths/min)	<18	18-23	24-29	≥30
Systolic blood pressure (mmHg)	<140	140-159	160-179	≥180
Temperature (°C)	<37	37-37.9	38-38.9	≥39
Sweating (diaphoresis)	None	Mild	Moderate	Severe
Posturing during episodes (dystonia/decerebrate/decorticate)	None	Mild	Moderate	Severe

**Table 2:** Diagnosis likelihood tool (DLT) Maximum DLT score = 11; Paroxysmal Sympathetic Hyperactivity-Assessment Measure (PSH-AM) score [6] = CFS score + DLT score. PSH-AM score: <8 = PSH unlikely; 8-16 = PSH possible; ≥17 = PSH probable. Credit: Information in this table was derived from the original publication of the Paroxysmal Sympathetic Hyperactivity Assessment Measure (PSH-AM) score [6], published under the Creative Commons License.

Diagnostic features (1 point per feature)	Score
History of acquired brain injury	1
Clinical features occur simultaneously	1
Episodes are paroxysmal in nature	1
Episodes triggered by non-noxious stimuli (suctioning, repositioning)	1
Features persist for >3 consecutive days	1
Features persist for >2 weeks after brain injury	1
Episodes persist despite treatment of other causes	1
≥2 episodes per day	1
Absence of an alternative cause of features (sepsis, pain, drug withdrawal, etc.)	1
Features persist despite treatment of an alternative differential diagnosis	1
Medication administered to decrease sympathetic features (morphine, propranolol, clonidine, etc.)	1

The case files were examined for a period of two weeks, starting from the day PSH was suspected in the patient records, and any records with no clear mention of the score and a PSH-AM score below 16 were not included. Patients' response to treatment was tracked in terms of PSH-AM scores, and patients were followed till ICU discharge or death.

Data collection

Data was retrieved manually from the medical files obtained from the hospital record section, and investigations were noted from the Hospital Information System (HIS). The data included patient demographics and ICU severity score (Acute Physiology and Chronic Health Evaluation II (APACHE II) score) at admission. The date of primary neurological insult, which was either cardiac arrest or seizure after which the patient became unconscious, was noted, along with Glasgow Coma Scale (GCS) at admission, and the date the diagnosis of PSH was made by the clinician after the primary neurological insult. The trend of vital parameters (maximum value recorded during the 24 hour ICU chart), from the day PSH was suspected by the clinician (from case files), including heart rate (HR), systolic blood pressure (SBP), respiratory rate (RR), temperature, and their response after initiation of therapy for PSH were analyzed with the PSH-AM score, including CFS and DLT for a period of two weeks at different points: at the time of diagnosis of PSH, two days, seven days and 14 days, respectively. Patients who had hemorrhagic shock or any form of active infection, such as catheter-associated urinary tract infection, ventilator-associated pneumonia, or bloodstream infection, during the study period were excluded from the analysis. The recorded outcome measures included the length of stay in the intensive care unit (LOS ICU), GOS-E at discharge, and mortality rate within 30 days.

Data representation and statistical analysis

Categorical variables were displayed as numbers and percentages, while continuous variables were assessed for normality. Because the data were non-parametric, they were shown as the median along with the interquartile range. The repeated measures Friedman test was applied to compare continuous variables such as HR, SBP, RR, duration of fever, and PSH-AM score at diagnosis and following treatment initiation at various previously described time points. A p-value of less than 0.05 was interpreted as indicating statistical significance. IBM SPSS Statistics for Windows, Version 21 (Released 2012; IBM Corp., Armonk, New York, United States) was used for data analysis.

## Results

**Figure 1:**
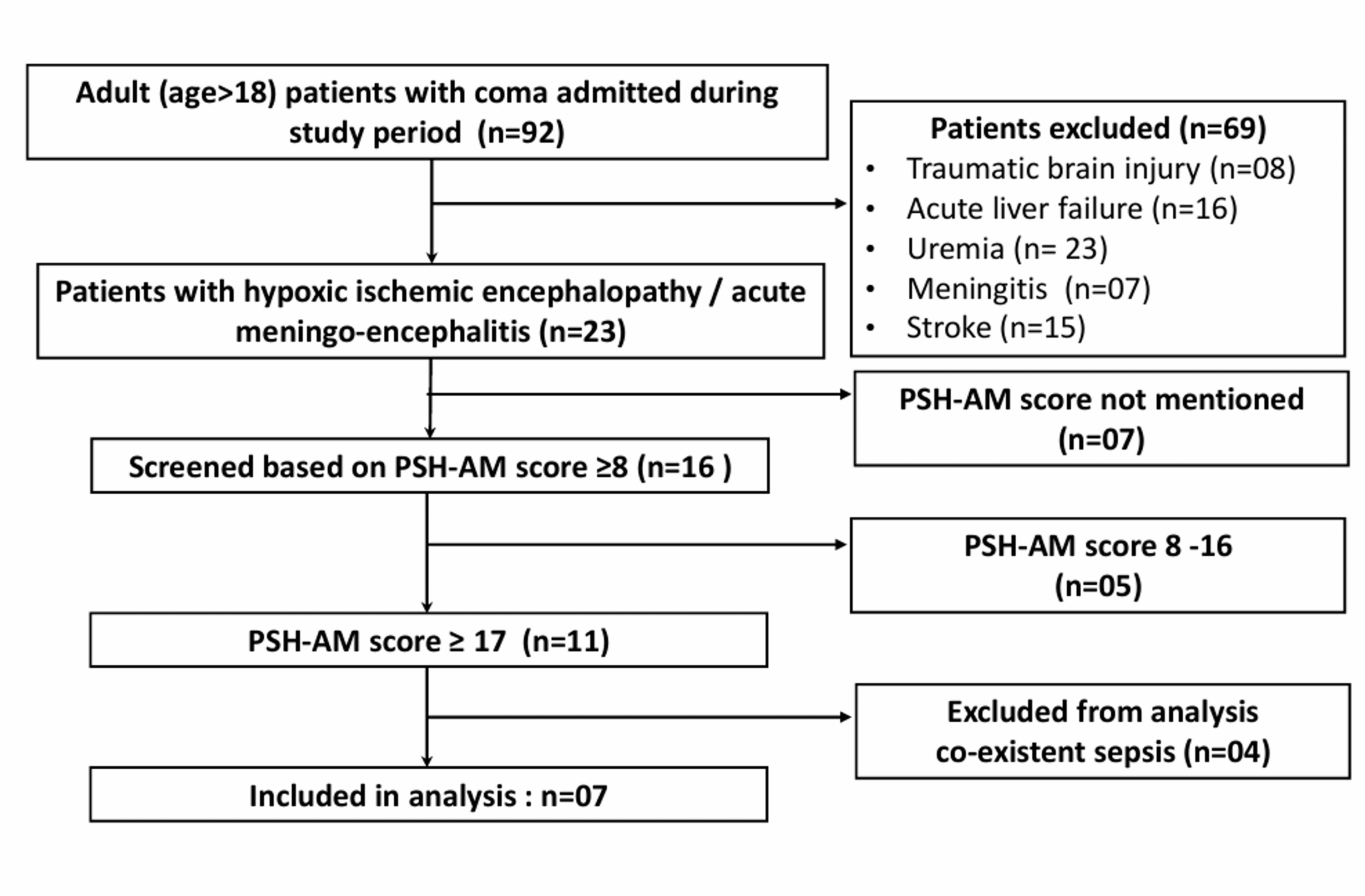
Flow diagram of the study PSH-AM: paroxysmal sympathetic hyperactivity-assessment measure

During the study period, 92 adult patients with coma were admitted to the ICU. Of these, 23 patients (25%) met the inclusion criteria while 69 patients were excluded from the analysis. Out of 23 patients, seven patients were excluded because of improper documentation of the PSH-AM score. A total of 16 patients (17.4%) were noted as having a presumed diagnosis of PSH (PSH-AM score ≥8). There were five patients out of 16 (31.3%) with a PSH-AM score of 8-16. There were 11 patients (68.8%) with a PSH-AM score of 17 or higher; four were excluded due to active infection during the study period, and finally, seven patients were included in the study (Figure 1).

The study analyzed patient records of seven patients with a diagnosis of probable PSH (PSH-AM score ≥17). The median age of the study cohort was 41 years (35-48 years), with 57.14% of them being males. The median value of severity of illness at ICU admission as assessed by APACHE II was 19 (13-21). Other baseline characteristics of the patients are represented in Table 3.

**Table 3:** Baseline characteristics of the patients (n = 7) IQR: interquartile range, BMI: body mass index, APACHE-II: Acute Physiology and Chronic Health Evaluation-II, GCS: Glasgow Coma Scale, PSH: paroxysmal sympathetic hyperactivity, PCT: procalcitonin, TLC: total leucocyte count

Characteristics	Median (IQR)/n (%)
Age (years)	41 (35-48)
Sex (male)	4 (57.1)
BMI (kg/m^2^)	23 (21-25)
Diagnosis at ICU admission: post-cardiac arrest hypoxic-ischemic encephalopathy (n)	3 (42.9)
Diagnosis at ICU admission: acute encephalitis (n)	4 (57.1)
APACHE II (at ICU admission)	19 (13-21)
Mechanical ventilation	7 (100)
Vasopressor support	4 (57.1)
Renal replacement therapy	1 (14.3)
GCS: motor component	3 (1-3)
Days of presumed neurological insult to the PSH diagnosis	38 (21-42)
PCT (ng/mL) at PSH diagnosis	0.16 (0.14-0.23)
TLC (10^3^/cu.mm) at PSH diagnosis	10.9 (07-12.5)

The median day of onset of PSH from the day of neurological insult was found to be 38 days (21-42 days). The median HR at the time of diagnosis was 128/minute (127-142), which significantly reduced to 102/minute (94-104) after two weeks of treatment (p < 0.001). The median SBP at the time of diagnosis was 142 mmHg (130-148), which significantly reduced to 118 mmHg (110-132) after two weeks of treatment (p < 0.002). The median RR at the time of diagnosis was 31/minute (27-32), which significantly reduced to 22/minute (22-28) after one week of treatment (p < 0.001). The median hours of fever duration at the time of diagnosis were 7 (6-10), which significantly reduced to two hours (0-6) after one week of treatment (p < 0.001). The median PSH-AM score was 20 (18-21) at the time of diagnosis, which was significantly reduced to 5 (3-14) after two weeks of treatment (p < 0.001) (Table 4).

**Table 4:** Trends of clinical characteristics and PSH-AM score The Friedman test was done due to a small sample size and non-normal distribution of data. The test does not assume sphericity and is appropriate for ordinal or non‑parametric repeated measures. The Friedman test indicated a significant difference across four time points, χ^2 ^(df = 4-1 = 3), p < 0.05. The chi-square statistic and degree of freedom are mentioned in the table. A p-value <0.05 was considered significant. PSH: paroxysmal sympathetic hyperactivity, SBP: systolic blood pressure, PSH-AM: Paroxysmal Sympathetic Hyperactivity-Assessment Measure

Characteristic (median with IQR)	At PSH diagnosis	Two days	One week	Two weeks	Chi-square statistic (df)	p-value
Heart rate (per minute)	128 (127-142)	125 (120-128)	110 (102-118)	102 (94-104)	23.4 (3)	<0.001
SBP (mmHg)	142 (130-148)	132 (120-142)	132 (120-136)	118 (110-132)	17.5 (3)	<0.002
Respiratory rate (per minute)	31 (27-32)	29 (24-30)	22 (22-28)	22 (20-22)	21.5 (3)	<0.001
Duration of fever (hours)	7 (6-10)	6 (3-10)	2 (0-6)	0	17.0 (3)	<0.001
PSH-AM score	20 (18-21)	19 (18-20)	15 (7-17)	5 (3-14)	22.5 (3)	<0.001

**Figure 2:**
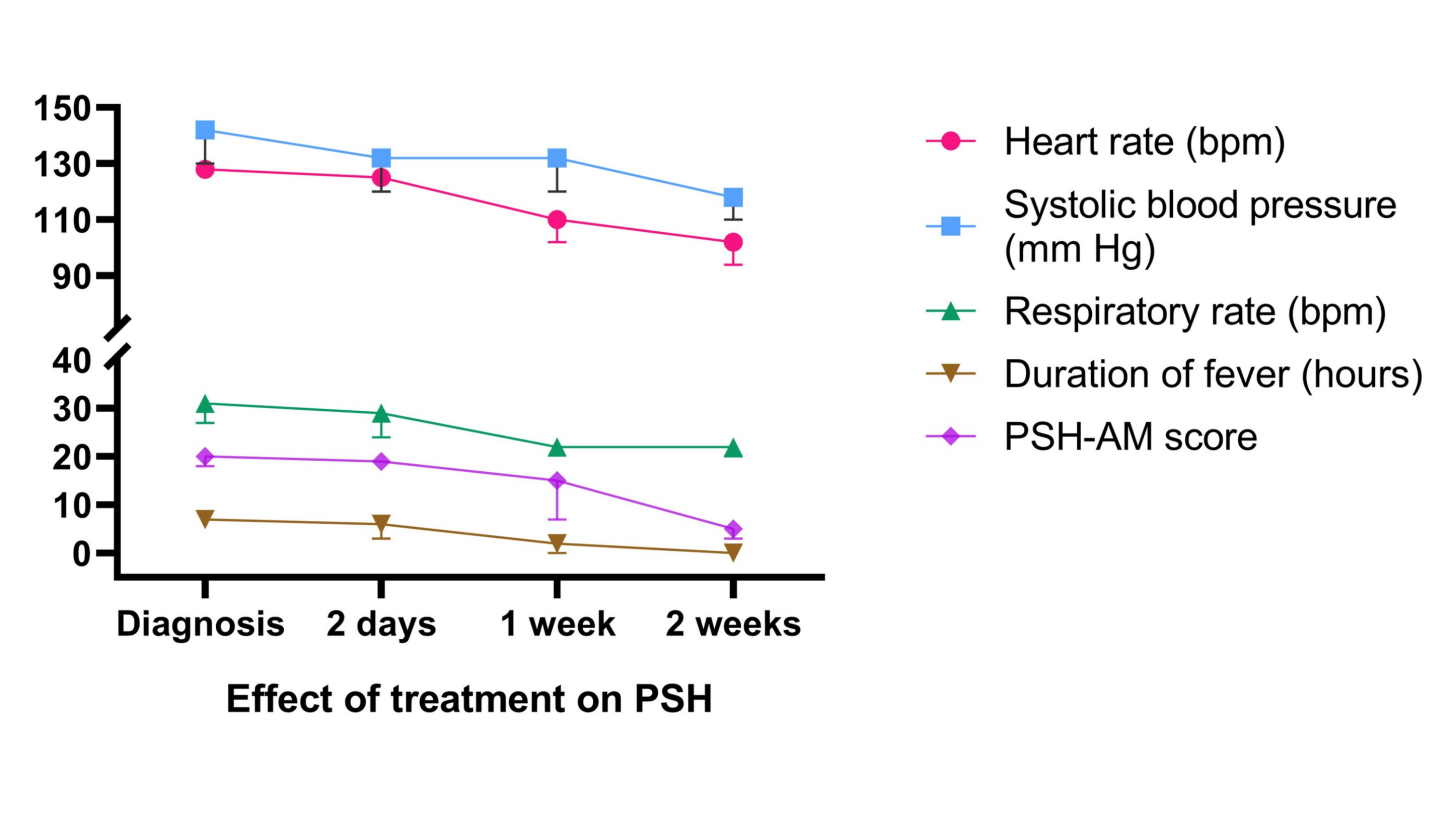
Trends of heart rate, systolic blood pressure, respiratory rate, duration of fever (hours) and PSH-AM score after initiation of therapy PSH: paroxysmal sympathetic hyperactivity, PSH-AM: Paroxysmal Sympathetic Hyperactivity-Assessment Measure

The medical interventions done at the diagnosis of PSH included intravenous fluid boluses (85%), benzodiazepine (midazolam) (43%), opioid (fentanyl) (71%), anti-pyrectic (paracetamol) for fever episodes (100%), passive cooling measures such as cooling mattress usage (29%), anti-epileptics (71%), changing existing invasive lines (29%) and empirical anti-microbials (100%) of patients. Mechanical ventilation for tachypnoea was given in all the study cases. After initiation of central alpha-2 agonists (clonidine) and non-selective beta-blockers (propranolol), the requirement for such additional supportive measures reduced. The prescription pattern of propranolol and clonidine as per the clinical trajectory of the patients diagnosed with PSH is mentioned in the Appendices. The effect of treatment on the hemodynamic parameters, i.e., HR, SBP, RR, and the duration of fever, along with response to therapy, is represented in Figure 2.

The patients were on prolonged ventilation support for 23 (14-32) days, till the diagnosis of PSH, and they were liberated from mechanical ventilation 13 days (10-24) after the therapy was initiated. One patient succumbed to his illness due to septic shock after 40 days of diagnosis of PSH, while the remaining six patients were discharged from the ICU. Other outcome parameters, such as the GOS-E at discharge, LOS ICU, are mentioned in Table 5.

**Table 5:** Outcome data of the patients IQR: interquartile range, PSH: paroxysmal sympathetic hyperactivity, GOS-E: Glasgow Outcome Scale Extended, LOS ICU: length of stay in intensive care unit

Characteristics	Median (IQR)/n (%)
Duration of mechanical ventilation (days)	53 (31-66)
Liberation (in days) from mechanical ventilation from the start of treatment of PSH (n = 6)	13 (10-24)
LOS ICU (days)	81 (71-104)
Outcome: survivors	6 (85.7)
GOS-E at discharge: category - 1 (dead)	1 (14.3)
GOS-E at discharge: category - 2 (vegetative state)	4 (57.1)
GOS-E at discharge: category - 3 (severe disability)	1 (14.3)
GOS-E at discharge: category - 4 (moderate disability)	1 (14.3)

## Discussion

This retrospective study observed a prevalence of 17% of probable PSH among comatose patients with hypoxic brain injury and encephalitis. This finding highlights that PSH is not uncommon in this population and may be underrecognized in routine ICU practice. Most existing literature focuses on TBI cohorts, where the reported incidence ranges from 10% to 30%. Our findings extend the current understanding by demonstrating that non-traumatic etiologies may also carry a significant risk of PSH, emphasizing the need for heightened clinical vigilance. Its diagnosis is important as it might lead to delayed weaning from mechanical ventilation, excessive polypharmacy (antibiotics, sedatives, anti-pyrectics, anti-epileptics), and thereby prolonging the LOS ICU.

A notable finding in our study was the delayed onset of PSH, with a median time of 38 days from the primary neurological insult. This contrasts with TBI, where PSH typically develops within three to 14 days [10,11]. Several factors may explain this delay. Patients with hypoxic brain injury often receive prolonged sedation, neuromuscular blockade, and targeted temperature management, which may mask early clinical manifestations. Additionally, delayed neuronal injury and progressive disruption of autonomic pathways may contribute to late onset [12]. This observation has important clinical implications, as PSH may not be suspected beyond the early ICU period, potentially leading to delayed diagnosis. The pathophysiology of PSH remains incompletely understood; however, the most widely accepted mechanism involves the disruption of inhibitory cortical pathways that regulate sympathetic activity. In hypoxic brain injury, diffuse cortical and subcortical neuronal injury disrupts widespread autonomic networks [13,14]. Similarly, in encephalitis, inflammatory involvement of cortical and subcortical structures may impair autonomic regulation [15]. These mechanisms contribute to an imbalance between excitatory and inhibitory pathways, often described as the EIR model, resulting in episodic sympathetic storms [12,16].

Diagnosing PSH in critically ill patients is challenging due to the presence of multiple mimickers such as sepsis, seizures, drug withdrawal, pain, and metabolic disturbances. In our study, alternative diagnoses were systematically excluded using clinical evaluation, laboratory parameters including procalcitonin and total leukocyte count, and electroencephalography where appropriate. ICU patients can be "mislabelled" as having "culture-negative sepsis,” with PSH being the hidden entity [17]. The episodic nature of symptoms and their association with triggering stimuli, such as suctioning and repositioning, were key features supporting the diagnosis of PSH. The use of the PSH-AM score provided an objective and standardized approach to diagnosis. PSH-AM score, used in this study, has a sensitivity of 94% and a specificity of 35% when used retrospectively in previous studies on TBI [11]. All patients included in our study had PSH-AM scores ≥17, consistent with probable PSH. The median PSH-AM score at diagnosis was 20, which significantly decreased to five after two weeks of treatment (p < 0.001), indicating both diagnostic validity and utility in monitoring response to therapy. CFS may be persistently elevated in hypoxic ischemic encephalopathy due to the global nature of the insult [6]. While PSH-AM has been reported to have high sensitivity but limited specificity, its use in conjunction with careful clinical assessment improves diagnostic confidence [13].

PSH was associated with a significant clinical burden in our cohort. Patients required prolonged mechanical ventilation, with a median duration of 53 days, and had extended ICU stays with a median of 81 days. The presence of PSH can complicate ICU management by delaying ventilator weaning, increasing sedation requirements, and predisposing to secondary complications such as infections and metabolic disturbances. Furthermore, misdiagnosis may lead to diagnostic errors, which can lead to unnecessary use of antibiotics, antiepileptic drugs, and investigations, thereby increasing healthcare costs and patient morbidity [18].

Management of PSH remains largely supportive and focuses on controlling sympathetic overactivity and minimizing triggering stimuli [19]. In our study, therapy was done with beta blockers (propranolol) and alpha-2 agonists (clonidine). This is consistent with existing literature, which suggests that combination therapy targeting multiple pathways is often required for effective symptom control [6,12,19]. Treatment was associated with significant improvement in physiological parameters, including HR, blood pressure, RR, and duration of febrile episodes, along with a marked reduction in PSH-AM scores over time.

Despite the severity of illness, the survival rate in our cohort was relatively high (85.7%). However, neurological outcomes remained poor in many patients, as reflected by low GOS-E at discharge. This suggests that while PSH is a treatable condition, it is often a marker of severe underlying brain injury and may not independently determine long-term neurological recovery [3,6,13].

Limitations

The major limitation of the study is the retrospective design, which would have missed cases with hidden PSH-like activity and the effect of recall bias. The scores could have been lower, as subjective components like sweating and posturing would not have been properly evaluated. Apart from this, the study is limited by its reproducibility due to a small sample size (n = 7) and a lack of standardized treatment protocols. The outcome of cases with PSH-AM scores less than 16 was not evaluated. There was no uniform treatment for those cases with a PSH-AM score ≥17, and the analysis was done on a very small number of patients (n = 7). Scores of 8-16 were unevaluated, resulting in missed diagnoses for these patients, which could further affect the generalizability. An audit on the excessive use of drugs, organ supports, changing existing invasive catheters, and investigations during the period of PSH, assuming it to be a seizure or infection, could have further highlighted the hidden cost of underdiagnosis of PSH in the study.

Strengths

The study addresses an underexplored area, PSH in non‑TBI, whereas most literature focuses on TBI. Early identification and appropriate management can reduce symptom burden, prevent unnecessary interventions, and potentially improve ICU outcomes. Clinicians should maintain a high index of suspicion for PSH in patients with recurrent, unexplained episodes of autonomic instability, especially when these episodes are paroxysmal and triggered by external stimuli. The study adds to the literature that PSH should be screened in patients with vegetative state/coma in the ICU, as it can prolong the ICU stay and cost of therapy. This condition should be in the differentials, especially when the clinical condition is not in favor of culture-negative sepsis.

Future research

Further longitudinal studies with a prospective multi-center study design screening for PSH-AM score daily in patients with non-traumatic brain insult in the intensive care unit are needed to evaluate the impact of PSH in such a cohort. While the PSH-AM remains the gold standard, its low specificity in the ICU remains a challenge. Future modifications, such as incorporating automated physiological trending or re-weighting the DLT for non-traumatic etiologies, may enhance its utility in hypoxic and encephalitic populations [3]. The score should be revised for an ICU setting with less reliance on qualitative parameters like excessive sweating. Further clarity on how frequently this condition should be screened for should also be evaluated.

## Conclusions

A high index of suspicion should be kept for diagnosing PSH in patients with non-TBI, particularly those with hypoxic brain injury and encephalitis. Episodic tachycardia, hypertension, diaphoresis, and posturing are important features to suspect PSH in comatose patients with a history of non-TBI after ruling out other mimics in the ICU, such as sepsis. Early identification through the PSH-AM score can be helpful in such patients. Management with centrally acting sympatholytics like propranolol and clonidine can reduce the number of medical interventions. Due to the small sample and retrospective bias, this can be considered as preliminary evidence that requires confirmation through larger prospective clinical studies.

## Appendices

Prescription pattern of propranolol and clonidine as per the clinical course of the patients diagnosed with paroxysmal sympathetic hyperactivity (PSH)

**Table 6:** The prescription pattern of propranolol and clonidine as per the clinical trajectory of the patients diagnosed with PSH GOS-E: Extended Glasgow Outcome Scale, PSH: paroxysmal sympathetic hyperactivity, propranolol tablet in milligrams, clonidine tablet in micrograms, HIE: hypoxic ischemic encephalopathy, NA: not administered

Parameters	Case-1	Case-2	Case-3	Case-4	Case-5	Case-6	Case-7
Diagnosis at ICU admission	Post-cardiac arrest HIE	Post-cardiac arrest HIE	Acute encephalitis	Acute encephalitis	Acute encephalitis	Acute encephalitis	Post-cardiac arrest HIE
At PSH diagnosis: Propranolol/Clonidine	NA/150	20/NA	NA/150	20/NA	NA/100	20/NA	40/NA
At 48 hours: Propranolol/Clonidine	20/300	30/NA	20/300	30/NA	NA/150	20/200	40/150
At 7 days: Propranolol/Clonidine	20/300	30/NA	40/300	30/NA	NA/ 200	40/200	60/150
At 14 days: Propranolol/Clonidine	20/300	30/NA	40/300	30/NA	NA/200	60/300	60/300
Outcome: survivor/non-survivor	survivor	survivor	survivor	survivor	survivor	survivor	non-survivor
GCS (discharge)	E4V2M4	E4VTM4	E4VTM4	E4V5M6	E4VTM2	E4VTM4	Death
GOS-E at discharge (category)	3	2	2	4	2	2	1
